# Human papillomavirus detection using the Abbott RealTi*m*e high-risk HPV tests compared with conventional nested PCR coupled to high-throughput sequencing of amplification products in cervical smear specimens from a Gabonese female population

**DOI:** 10.1186/s12985-017-0906-2

**Published:** 2017-12-21

**Authors:** Pamela Moussavou-Boundzanga, Ismaël Hervé Koumakpayi, Ingrid Labouba, Eric M. Leroy, Ernest Belembaogo, Nicolas Berthet

**Affiliations:** 1 Centre International de Recherches Médicales de Franceville (CIRMF), Department of Zoonosis and Emerging Diseases, 769 Franceville, BP Gabon; 2 Institut de Cancérologie de Libreville (ICL), Libreville, Gabon; 3 Institut de Recherches et de Développement (IRD), Maladies Infectieuses et vecteurs : Ecologie, Génétique, Evolution et Contrôle (IRD 224 – CNRS 5290 6 UM1- UM2), Montpellier, France; 4 Centre National de Recherche Scientifique (CNRS), UMR3569, 25 rue du docteur Roux, 75724 Paris, France; 5 Institut Pasteur, Unité Environnement et risques infectieux, Cellule d’Intervention Biologique d’Urgence, 25 rue du Docteur Roux, 75724 Paris, France

**Keywords:** Abbott RealTi*m*e high-risk HPV, Conventional PCR, Human papillomavirus, Screening, Cervical cancer

## Abstract

**Background:**

Cervical cancer is the fourth most common malignancy in women worldwide. However, screening with human papillomavirus (HPV) molecular tests holds promise for reducing cervical cancer incidence and mortality in low- and middle-income countries. The performance of the Abbott RealTi*m*e High-Risk HPV test (AbRT) was evaluated in 83 cervical smear specimens and compared with a conventional nested PCR coupled to high-throughput sequencing (HTS) to identify the amplicons.

**Results:**

The AbRT assay detected at least one HPV genotype in 44.57% of women regardless of the grade of cervical abnormalities. Except for one case, good concordance was observed for the genotypes detected with the AbRT assay in the high-risk HPV category determined with HTS of the amplicon generated by conventional nested PCR.

**Conclusions:**

The AbRT test is an easy and reliable molecular tool and was as sensitive as conventional nested PCR in cervical smear specimens for detection HPVs associated with high-grade lesions. Moreover, sequencing amplicons using an HTS approach effectively identified the genotype of the hrHPV identified with the AbRT test.

## Background

Infections with human papillomavirus (HPV) are the most common sexually transmitted infections worldwide [1]. Persistent cervical infection is a high risk factor for cervical cancer, which is the fourth most common malignancy in women worldwide. Moreover, there is a clear etiological link between the persistence of HPV infections in epithelial cells of the cervical mucosa, precancerous lesions (i.e., cervical intraepithelial neoplasia 3; CIN3) and cervical cancer [2]. Today, more than 200 HPV genotypes have been defined, among which 50 can infect the cervical epithelia. However, only 16 HPV types (16, 18, 31, 33, 34, 35, 39, 45, 51, 52, 56, 58, 59, 66, 68, 70) are classified as high risk (hrHPVs) and 14 (excluding 34 and 59) show strong evidence of a causal link to cervical cancer [3].

Based on the clear causal link between hrHPVs and cervical cancer, the guidelines of the American Society for Colposcopy and Cervical Pathology recommend HPV DNA testing instead of cytology to carry out triage in a screened population. Molecular tests for HPV detection may be a good alternative to cytology for early screening because an HPV-negative result is sufficient to declare an extremely low risk of developing CIN3 or worse (CIN3+) for 5 years in contrast to a negative cytology result [4]. Other recent studies have confirmed that HPV type identification has significantly higher sensitivity than cytology [5]. Consequently, this higher sensitivity may be the reason behind an increase in the number of false positives, i.e. samples lacking high-grade lesions (CIN2+) [6]. Therefore, a single positive HPV test may be insufficient to identify women who run the risk of developing cervical neoplasia [7]. In response to this caveat, Meijer et al. (2009) published novel guidelines for assessing the performance of a new HPV DNA assay. Molecular assays must detect hrHPV infections that are preferentially associated with CIN2+, and thus they should have at least 90% sensitivity with GP5+/6+ primers or the Hybrid Capture 2 (hc2) test [8]. The hc2 test was approved by the United States Food and Drug Administration (FDA) as the gold standard for the detection of 13 hrHPV genotypes in 1999. However, this test has some disadvantages, including the non-discrimination of HPV16 or HPV18 from other hrHPVs and the absence of an internal control [7]. Furthermore, the hc2 test is associated with cross-reactivity of the probe mixture with untargeted HPV types [7]. To overcome this limitation, new HPV DNA testing assays (Abbott, Wiesbaden, Germany; LG Life Science, Seoul, Korea, etc.) based on real-time PCR, have been developed and can distinguish HPV16 or HPV18 from other major hrHPVs, and some are currently approved by the FDA for clinical use [7]. These molecular tests are recommended by the Guideline Development Group for the screen-and-treat strategy because their contribution may be greater than visual inspection with acetic acid and/or Lugol’s iodine (VIA/VILI) to reduce cervical cancer and its related mortality. Moreover, their implementation in low- and middle-income countries may be easier than cytology-based screening programs [9]. Furthermore, the quality of the molecular tests is not affected by most bacterial infections concomitant to the HPV infection. Finally, the level of technical training of the healthcare staff has only a minor impact on the performance of the test.

This study was based on the use of the Abbott RealTi*m*e High-Risk HPV (AbRT) assay for the detection of HPV in cervical cell samples of women with or without cervical abnormalities. The performance of the test was evaluated in regard to the detection of two main HPV genotypes, HPV16 and 18, and a panel of 12 hrHPVs, all constituting one single hrHPV category. HPV genotypes identified after sequencing amplicons generated by conventional nested PCR allowed us to determine the non-HPV16/non-HPV18 genotypes detected by the AbRT assay.

## Methods

### Study subjects and cervical sample collection

Participating women were selected from a 960 patient cohort previously constituted from March 2013 to January 2014 during a cervical lesion screening program as part of a multi-center cross-sectional study carried out in two hospitals located in Libreville, Gabon [10]. A total of 87 women met both main inclusion criteria, i.e. were aged 25 years or older and presented cervical abnormalities in a VIA/VILI test. Ultimately, 83 women were included in this study and collected specimens were divided into five groups according to cytological analysis: normal (group 1, *n* = 50), atypical squamous cells of undetermined significance (ASCUS, group 2, *n* = 12), low-grade squamous intraepithelial lesions (LSIL, group 3, *n* = 7), high-grade squamous intraepithelial lesions (HSIL, group 4, n = 7) and carcinoma (group 5, n = 7). Women infected by a low-risk HPV genotype — which cannot be detected by the AbRT assay — were excluded.

### DNA extraction from liquid-based cytology samples

DNA was extracted from exfoliated cells obtained from liquid-based cytology (LBC) using the DNeasy Blood and Tissue kit (Qiagen, Valencia, CA, USA) according to the manufacturer’s instructions. DNA was quantified using the Qubit® dsDNA BR Assay kit with the Qubit 2.0 fluorimeter (Life Technologies, Carlsbad, CA, USA) primarily to confirm the previous DNA purification step.

### Assays for HPV detection

**Fig. 1:**
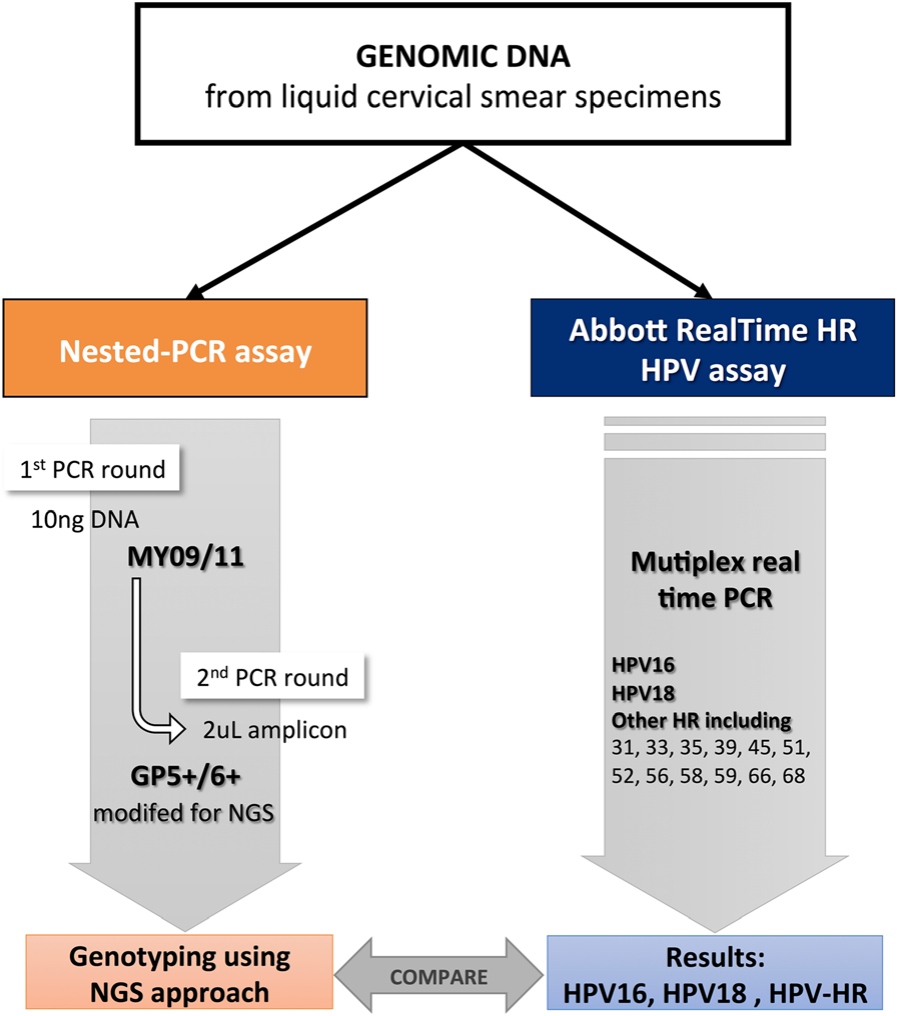
Comparison of Nested-PCR and Abbott Realtime HR-HPV

The AbRT assay (Abbott Molecular, Des Plaines, IL, USA) was performed with the *m*2000rt automated analyzer according to the manufacturer’s instructions. Detected HPVs were validated by genotyping a 150 bp fragment of the L1 HPV gene and discriminating between 14 hrHPVs: HPV16, HPV18 and a pool of 12 other hrHPVs (HPV 31, 33, 35, 39, 45, 51, 52, 56, 58, 59, 66 and 68). PCR amplification included 5 μL of the extracted DNA sample along with the GP5+/6+ primers designed for use with a high-throughput sequencing approach (HTS): HTS-GP5+: 5′ tcg-tcg-gca-gcg-tca-gat-gtg-tat-aag-aga-cag-TTG-TTA-CTG-TGG-TAG-ATA-CTA-C 3′; HTS-GP6+: 5′ gtc-tcg-tgg-gct-cgg-aga-tgt-gta-taa-gag-aca-gGA-AAA-ATA-AAC-TGT-AAA-TCA-TAT-TC 3′) and corresponding HPV probes [10] (the lowercase letters in the primer sequences are the Illumina adapter sequences added to the 5′ end of the original GP5+ and GP6+ primers [11] shown in uppercase letters). A 136 bp fragment of human β-globin gene was co-amplified as an internal control (Fig. 1).

In parallel, HPVs were also detected using an HTS approach based on the conventional HPV nested PCR. This PCR required both conventional MY09/11 primers (MY09, 5’-CGTCCMARRGGAWACTGATC-3′, and MY11, 5’-GCMCAGGGWCATAAYAATGG-3′, where M = A or C; R = A or G: W = A or T; Y=C or T [12]) and the HTS-adapted GP5+/6+ primers. For HPV nested PCR, 5 μL of each DNA sample was used for the first MY09/11 PCR and 2 μL of the MY09/11 PCR products was used for the second GP5+/6+ PCR. The sequencing of amplicons and the analysis of generated data were carried out as described previously [10] (Fig. 1).

### Statistics

Statistical analyses were performed using R software version 3.4.0. Cohen’s Kappa value, a measure of agreement, was used to evaluate the concordance between the two HPV detection methods used here. The Kappa statistic varies from 0 to 1, with unity indicating perfect agreement.

## Results

### HPV distribution among normal and dysplastic lesions with the AbRT assay and conventional nested PCR

The AbRT assay detected at least one HPV genotype in 44.57% of women (37/83). The mean age of the women who were positive for HPV DNA was 50 years. HPVs (genotypes 16 and/or 18 and/or other targeted hrHPVs) were identified in 26% (13/50) and 72.72% (24/33) of cases among women presenting normal and abnormal cytology, respectively. Moreover, the association between abnormal cytology and HPV infection was significant (*p* value˂0.001; odds ratio, OR = 8.89) and increased with the severity of the cervical lesions and the age of the patient (*p* < 0.05). Although 10.81% (4/37) of the women were infected by at least two different categories of HPV (16 and/or 18 and/or other hrHPVs), HPV16 was the most frequently detected genotype, followed by other hrHPVs and HPV18 with detection rates of 52.63%, 39.47% and 21.05%, respectively.

The two methods used in this study demonstrated their ability to detect at least one hrHPV genotype (HPV16 or HPV18 or other hrHPV or a mix of several hrHPVs) among 100% (14/14) of the HSIL+ cases tested (Table 1). The nested PCR method detected more HPVs (56.5% versus 33.3%) among HSIL- samples than the AbRT assay (Table 1).

**Table 1:** Comparison of HPV detection in high-grade lesions (HSIL+) between Abbott RealTi*m*e assay and conventional nested PCR

		HSIL+	
		**+**	**–**
Conventional nested PCR	**+**	14	39
	**–**	0	30
Abbott RealTi*m*e	**+**	14	23
	**–**	0	46

Among women presenting HSIL+ cervical abnormalities, HPV16 was detected by both methods in five cases of carcinoma and in five cases of HSIL (Table 2 and Table 3, cases no. 1 to 10). For two carcinoma cases (Table 3, cases no. 11 and 12), this genotype was only detected with the conventional nested PCR method. However, for these two cases, no discordant results for other detected genotypes (HPV18 and hrHPV for cases no. 11 and 12, respectively) were observed between the two methods. HPV18 was only detected with the AbRT assay in 50% (2/4) of cases in which it was identified with the nested PCR method (Table 2 and Table 3). For HSIL+ samples, the detection of genotypes HPV16 or HPV18 by both methods showed a moderate level of agreement of 85.71% with a Kappa value of 0.58 (Table 4).

**Table 2:** Positive detection rate for each of the two tested assays according to cytological results: normal, atypical squamous cells of undetermined significance (ASCUS), low-grade lesions (LSIL) and high-grade lesions + (HSIL+)

Assay	Normal (*n* = 50)	ASCUS (*n* = 12)	LSIL (*n* = 7)	HSIL+ (*n* = 14)
Conventional nested PCR	24 (48%)	6 (50%)	6 (85.71%)	14 (100%)
HPV16	21 (87.5%)	4 (66.67)	4 (66.67%)	12 (85.71%)
HPV18	5 (20.8%)	2 (33.33)	1 (16.67%)	3 (21.43%)
Other high-risk genotypes	15 (62.5%)	1 (16.67)	3 (50%)	4 (28.57%)
Abbott RealTi*m*e	13 (26%)	4 (33.33%)	6 (85.71%)	14 (100%)
HPV16	7 (53.85%)	2 (50%)	2 (33.33)	10 (71.43%)
HPV18	1 (7.69%)	2 (50%)	1 (16.67%)	2 (14.29%)
Other high-risk genotypes	7 (53.85%)	–	4 (66.67%)	3 (21.43%)

**Table 3:** Concordance between genotypes generated by conventional nested PCR (PCR GP) and the Abbott RealTi*m*e assay (AbRT) (P, positive; N, negative; hrHPV, high-risk HPV, a category including 12 different hrHPV genotypes)

Case number	PCR GP -HPV16	AbRT - HPV16	PCR GP -HPV18	AbRT - HPV18	PCR GP –hrHPV	AbRT - hrHPV	Cytology
1	P	P	N	N	N	N	carcinoma
2	P	P	N	N	N	N	carcinoma
3	P	P	N	N	N	N	carcinoma
4	P	P	N	N	N	N	carcinoma
5	P	P	N	N	N	N	carcinoma
6	P	P	N	N	N	N	HSIL
7	P	P	N	N	N	N	HSIL
8	P	P	N	N	N	N	HSIL
9	P	P	P	N	33	N	HSIL
10	P	P	N	N	33	P	HSIL
11	P	N	P	P	N	N	carcinoma
12	P	N	N	N	45	P	carcinoma
13	N	N	P	P	N	N	HSIL
14	N	N	P	N	33	P	HSIL
15	P	P	N	N	N	N	LSIL
16	P	P	N	N	N	N	LSIL
17	P	P	N	N	58	P	LSIL
18	P	P	N	N	N	N	LSIL
19	P	N	N	N	N	N	LSIL
20	P	N	N	N	33/32	N	LSIL
21	P	N	N	N	N	P	LSIL
22	P	N	N	N	N	P	LSIL
23	N	N	P	P	N	N	LSIL
24	N	N	P	P	N	N	LSIL
25	N	N	P	P	N	N	LSIL
26	N	N	N	N	33	P	LSIL
27	N	N	P	N	N	N	LSIL
28	P	N	N	N	31	N	normal
29	P	N	N	N	33	N	normal
30	N	N	N	N	33	P	normal
31	P	N	N	N	33	P	normal
32	N	N	N	N	35	P	normal
33	P	N	P	P	45	N	normal
34	N	N	N	N	58	N	normal
35	P	P	P	N	72	P	normal
36	P	N	P	N	33/35/45/58/72	N	normal
37	P	N	P	N	33/45/58/72	N	normal
38	P	N	N	N	33/45/72/81	P	normal
39	P	N	N	N	33/58	N	normal
40	P	P	P	P	33/72	P	normal
41	N	N	N	N	35/72	N	normal
42	P	N	N	N	45/72	P	normal
43	P	N	N	N	N	N	normal

**Table 4:** Agreement (%) between the Abbott RealTi*m*e and conventional nested PCR assays for the detection of the HPV16 and HPV18 genotypes among high-grade lesions (HSIL+) and low-grade lesions (LSIL)

		HSIL+	HSIL-
HPV16	Agreement	85.71	78.95
	Kappa coefficient	0.58	0.54
HPV18	Agreement	85.71	94.74
	Kappa coefficient	0.58	0.82

Regarding women with ASCUS, LSIL and normal cytology, HPV16 was detected in 14.5% (10/69) and 43.5% (30/69) of cases by the AbRT assay and the nested PCR method, respectively (Table 2). HPV18 was found in 10.1% (7/69) and 15.9% (11/69) of cases by the AbRT assay and the nested PCR, respectively (Table 2). Among all cases in which HPV16 and HPV18 were detected, only six and five cases were detected by both methods, respectively (Table 3). For LSIL, the two tested methods for detecting HPV16 and HPV18 showed moderate (78.95%) and high agreement (94.74%), with kappa values of 0.54 and 0.82, respectively (Table 4).

### Identification of hrHPV (non-HPV16 and non-HPV18) genotypes detected with the AbRT assay

Genotypes detected by the AbRT assay in the hrHPV category were compared with the results obtained from amplicons generated by the nested PCR method. The hrHPV category was detected in women presenting cytological abnormalities, with 14.28% (1/7), in 28.57% (2/7) and in 57.14% (4/7) of cases in carcinoma, HSIL and LSIL, respectively. Furthermore, the hrHPV category was also detected in 43.8% (7/10) of cases for women with normal cytology. Except for case no. 9, all HPV33 and HPV45 genotypes identified by PCR were also detected by the AbRT assay (Table 3, cases no. 10, 12 and 14) in HSIL+ samples. In case no. 9, HPV33 was only detected after sequencing the amplicon (Table 3).

Among all the LSIL samples tested, four samples tested positive with the AbRT assay, but only two (cases no. 17 and 26) were identified by both methods. Two cases (no. 21 and 22) were not identified by nested PCR, whereas for case no. 20, the AbRT assay failed to detect HPV33, which was identified by nested PCR. Among samples with normal cytology, at least one hrHPV genotype was detected in 94.7% (18/19) of cases by nested PCR (Table 3). However, in only 38.9% of cases (7/18), the AbRT assay also indicated a positive signal for hrHPV. Unlike previous results for LSIL+ samples in which only one hrHPV genotype was detected each time, at least two hrHPV genotypes were detected in 22% of cases with normal cytology (Table 3). Finally, only the result for case no. 35 was discordant: the low-risk HPV72 genotype identified by nested PCR is not included in the hrHPV category detected with the AbRT assay.

## Discussion

This study describes the first use of the Abbott RealTi*m*e High-Risk HPV assay for HPV detection in cervical cell samples of women with or without cervical abnormalities from a Gabonese female population. Moreover, this assay was compared with the use of conventional nested PCR coupled to HTS, which identifies the exact hrHPV genotypes revealed with the AbRT assay. The real-time PCR method (AbRT assay) can detect a total of 14 different high-risk HPV genotypes whereas the conventional nested PCR that uses the primers MY09/11 and GP5+/6+ can amplify around 30 different low- and high-risk HPV genotypes [13, 14]. The genotypes are assayed from DNA extracted from cervical smears whose cervical anomalies have been revealed by a VIA/VILI test and confirmed by cytology. Although the manufacturer recommends using the Abbott automaton (*m*200sp, Abbott Molecular) with this assay for HPV detection, extractions were performed manually as described in literature as a practicable alternative. In a recent study, Kocjan et al. (2012) showed that similar results are obtained for HPV detection from biological tissues of head-neck squamous cell cancers regardless of the extraction method used with the AbRT assay [15, 16]. Moreover, we did not observe any differences in amplification intensity of the human β-globin (a housekeeping gene) between the AbRT kit and a classic qPCR protocol (data not shown).

In this study, the AbRT generally gave consistent genotyping results for the hrHPV screened in HSIL+ cases in comparison with the conventional nested PCR. These data are similar to other studies that compared the AbRT kit with this nested PCR method or with other molecular methods of HPV genotyping [15, 16]. However, nested PCR appears to be more sensitive than the AbRT kit for the detection of hrHPV in HSIL- cases (i.e. LSIL, ASCUS and normal) even if in most cases, at least one hrHPV genotype was detected by both methods (Table 3). Given that the detection threshold of the AbRT assay is between 500 and 5000 genome copies, genotypes detected using both methods in this study may have a higher number of copies than this threshold [17], suggesting that cases for which only one genotype was detected by the nested PCR method contain fewer than 500 copies. However, this hypothesis remains to be tested because we have no data on the actual viral loads of our samples. Furthermore, although the correlation between the number of reads and viral load is not perfect, cases with genotypes detected by both methods were generally those with highest numbers of sequenced reads [10]. Owing to the depth of sequencing with a minimum of several hundred thousand reads per sample, a minor HPV genotype can be detected if it is represented in at least 2% of the total reads [10]. Therefore, in a given sample, HTS can unambiguously detect several genotypes whose relative proportions potentially vary [18, 19]. Moreover, although the sequenced amplicon size was only 150 bp, this region of the L1 gene is highly amenable to molecular analysis, which can discriminate between the different HPV genotypes. Finally, the HTS approach for sequencing HPV amplicons may overcome the limit of direct amplicon sequencing using the Sanger method, which is simple to implement from a biopsy, but cannot be performed on DNA extracted from a (LBC) cervical smear, especially when the cytological grade is low or normal, due to the potential simultaneous presence of several HPV genotypes [20]. Although HPV genotyping assays using consensus or broad-spectrum primers are less sensitive than type-specific or targeted primers [21], consensus PCR is less affected by competitive primer binding in mixed HPV infections. For instance, the presence of high viral loads of HPV16 or, in some cases, the presence of some hrHPVs can mask the HPV52 genotype in anogenital samples [22] or inhibit the amplification and detection of HPV31 and 33 [23]. Moreover, unfavorable amplification kinetics may occur when an HPV genotype belonging to the alpha-9 subgenus is in the presence of other genotypes (Iftner et al. (2016).

Several studies have shown that the sensitivity of the AbRT assay is similar to that of the hc2 test and that the specificity of detection of HPVs associated with the development and/or spread of CIN2+ can reach 92% [16, 24, 25]. GP5+/6+ PCR has the same clinical performance as the hc2 test [8]. However, the addition of the first nested PCR step with MY09/11 primers improves the sensitivity of the conventional PCR test, but leads to a decrease in its specificity for the detection of HPV genotypes shown to be associated with the development of HSIL+ into cancer [26]. Furthermore, the nature of the samples used in this study clearly influences the differences observed between the two methods. In contrast to samples from biopsies, LBC samples recover numerous superficial cells in addition to the cells located in a putative precancerous lesion. However, these superficial cells may be infected by the same hrHPV genotype or other hrHPV genotypes. The use of a sensitive HPV detection method may reveal these supplementary infections and not just the HPV implicated in the (pre)cancerous lesion. In effect, due to this high sensitivity, most studies on the clinical validation of HPV detection tests are carried out on FFPE tissue blocks, not LBC samples, and cytological results are generally confirmed by a histological analysis to confirm the grade of the observed lesion. Therefore, the differences between the two molecular methods for HPV detection are most likely due to the nature of the samples used in this study and may have led to variation in the performance of the test and the specificity of the AbRT compared with the data found in literature [15, 24, 27–29]. However, the use of LBC led to a better preparation of the sample associated with an enhanced quality of the cytological results. Recent studies have shown improved slide reading when LBC systems are used instead of conventional Pap smears in a routine clinical setting. Moreover, LBC using PreservCyt (Thinprep) has been approved by the FDA for molecular HPV tests [30]. This FDA approval provides the opportunity to carry out two biological analyses from a single sample, a certain advantage in low-income countries or when the population has limited access to healthcare.

## Conclusions

This study on the performance of the AbRT assay shows that it is an easy and reliable molecular tool for HPV detection in HSIL+ LBC samples. However, as expected, the AbRT assay is less sensitive than conventional nested PCR for the detection of HPV in HSIL-. Moreover, the sequencing of amplicons using a HTS approach effectively identified the genotype of hrHPV detected with the AbRT assay. Finally, our data showed that the use of AbRT in co-testing with VIA/VILI in low-income countries holds promise as an interesting alternative to other combinations, such as VIA and cytology.

## Abbreviations

AbRT: Abbott RealTime
ASCUS: Atypical squamous cells of undetermined significance
CIN: Cervical intra-epithelial neoplasia
FDA: Food and Drug Administration
FFPE: Formalin-fixed paraffin-embedded
HPV: Human papillomavirus
hrHPV: high-risk human papillomavirus
HSIL: High-grade squamous intraepithelial lesion
HTS: High-throughput sequencing
LBC: Liquid-based cytology
LSIL: Low-grade squamous intraepithelial lesion
VIA: Visual inspection with acetic acid
VILI: Visual inspection with Lugol’s iodine
